# Sport Participation and Psychosocial Factors Which Influence Athletic Identity in Youth Athletes With Anterior Cruciate Ligament Injury

**DOI:** 10.3389/fpsyg.2022.906300

**Published:** 2022-05-31

**Authors:** James McGinley, Emily Stapleton, Hannah Worrall, Henry B. Ellis, Philip L. Wilson, Sophia Ulman

**Affiliations:** ^1^Department of Biomedical Engineering, The University of Texas at Austin, Austin, TX, United States; ^2^Center for Excellence in Sports Medicine, Scottish Rite for Children Orthopedic and Sports Medicine Center, Frisco, TX, United States; ^3^Department of Psychology, Scottish Rite for Children, Frisco, TX, United States; ^4^Department of Orthopaedic Surgery, University of Texas Southwestern Medical Center, Dallas, TX, United States

**Keywords:** athletic identity measurement scale, adolescent, return to sport, psychology, rehabilitation, athletic identity

## Abstract

Athletic identity, or the degree with which individuals identify with the athlete role, is an important rehabilitation factor for sports care providers to consider; however, it lacks extensive study in youth. The purpose of this study was to identify demographic, sport participation, and psychosocial measures which correlate with youth athletic identity after anterior cruciate ligament injury. Participants completed standardized sports medicine intake and patient-reported outcome measures, including the Athletic Identity Measurement Scale (AIMS). A total of 226 participants were included, and two groups were created based on high or low total AIMS score. Results indicated that sex (*p* = *0.002*), years active in sport (*p* = *0.049*), activity level (*p* = *0.038*), and ACSI-Coachability (*p* = *0.027*) differed by AIMS score. While youth athletes appear resilient, these results emphasize that they identify strongly with the athlete role and may suffer psychosocial consequences after injury. Future work should evaluate similar factors over course of recovery in a larger, diversified population.

## Introduction

Sports are an integral part of life for many children. Youth athletes’ involvement in sport varies – for some it is the sole basis of friendships and recreation, for others it is merely a hobby (McKay et al., 2019). Sports may confer strong benefits to the participant in the avenues of physical health, teamwork development, and mental improvement (Panza et al., 2020). However, participation in sport for elite athletes may result in time commitments that conflict with other aspects of life, such as quality time for peer connections and academics (Eime et al., 2013). Furthermore, injuries may occur in both elite and recreational athletes which result in loss of the benefits and identity of sports participation and in dedicating a significant amount of time to injury rehabilitation (Christino et al., 2015). Therefore, a child’s integration and involvement with sport may be a primary determinant of overall well-being, and identification of this association may assist health care providers when planning treatment strategies and/or anticipating response to treatment. This strong relationship with sport may be characterized as athletic identity, first defined by Brewer et al. (1993) as the degree with which individuals identify with the athlete role.

The association of athletic identity and specific demographic characteristics is poorly described in the literature. Some studies suggest that sex is significantly correlated with athletic identity in youth (Anderson et al., 2009) and young adults (Babić et al., 2015; Şekeroğlu, 2017). Yet, others claim there is no correlation with athletic identity in youth (Padaki et al., 2018; Piatt et al., 2018). In young adults, Lamont-Mills and Christensen (2006) saw no correlations with athletic identity, while Proios et al. (2012) observed differences only in the subcategory of how exclusive athletes were to their athletic identity over other identities. Association of athletic identity and race or ethnicity in the youth population is also poorly defined, though Anderson et al. and others suggest that Hispanic and minority youths perceive themselves as having lower athletic identity (Anderson et al., 2009, 2011; Edison et al., 2021). Youth athletes appear to conflate athletic identity and personality as they mature (Von Rosen et al., 2018; Newton et al., 2020; Edison et al., 2021), though athletic identity may begin to decrease with age at the undergraduate level (Renton et al., 2021). The convergence of athletic identity and personality in youth may prove problematic if their athletic identity is interrupted. Upon transition out of sport, being cut from a team, or experiencing an injury, athletes may feel a major aspect of their personality is unfulfilled, especially as single-sport specialization becomes more common with increasing age (Jayanthi et al., 2013).

Similarly, athletic identity and sport participation are not clearly related; however, the literature suggests increased training hours may significantly increase athletic identity (Piatt et al., 2018; Quinaud et al., 2020). Conversely, there has been conflicting data regarding athletic identity as a driving determinant of activity level (Reifsteck et al., 2013; Ohji et al., 2021). Ultimately, the relationship of athletic identity and patient-reported sport participation measures is unclear (Ardern et al., 2014).

Coping and fear of re-injury are likely associated with athletic identity, though the available literature is limited. Emotional trauma and depressive symptoms have been shown to increase with greater athletic identity in youth samples after injury (Manuel et al., 2002; Padaki et al., 2018). Additionally, McKay et al. (2013) noticed differences in injury patterns after youth ice hockey players underwent anterior cruciate ligament (ACL) injury.

There are unclear associations between athletic identity and demographics or psychological readiness within the spectrum of recreational to elite youth athletes due to the overall sparse literature. As such, sports care providers may have inadequate insight on psychological responses to injury and treatments within the adolescent population with differing levels of athletic identity. Therefore, the purpose of this study was to identify demographic, sport participation, and psychosocial measures associated with athletic identity in a youth athlete population after anterior cruciate ligament injury.

## Materials and Methods

This study was approved by the local Institutional Review Board (IRB; #STU-2019-0701). Informed consent was not required and was waived by the IRB as this study only consisted of a retrospective review of previously collected data.

### Participants

A consecutive review of participants between the ages of 10.5 – 24.9 years (mean 15.9 years) who presented to a pediatric sports medicine practice and completed the standardized sports medicine intake and patient-reported outcome (PRO) measures between October 2019 and May 2021 was conducted. All participants were included if they completed the pre-visit intake questionnaire before the rehabilitation process including the Athletic Identity Measurement Scale (AIMS), a self-report measure used to quantify athletic identity. All participants (N = 226) presented with anterior cruciate ligament injury and were reflective of the study region in race and ethnicity. Participants were evenly distributed between male and female and competed predominantly in a single sport (57.8%) at the school level (58.2%; Table 1).

**TABLE 1:** Participant demographics and sport characteristics.

Variable	Group	N (%)
*Sex*	Female	115 (50.9)
	Male	111 (49.1)
*Race*	White	150 (66.7)
	Black or African American	52 (23.1)
	Non-White or Black	23 (10.2)
*Ethnicity*	Hispanic or Latino	64 (28.6)
	Non-Hispanic or Latino	160 (71.4)
*Competition Level*	School	82 (58.2)
	Select/Club	47 (33.3)
	College	3 (2.1)
	Other	9 (6.4)
*Sport Participation*	Single-Sport	93 (57.8)
	Multi-Sport	60 (37.3)

*Non-White or Black may include Native American or Alaskan Native, Indian, Asian, or mixed race. Competition level was reported as the highest level achieved, with School as the lowest level and College as the highest level. Other competition levels may be described as recreational or non-competitive.*

### Procedures

Data was collected from participants at the initial pre-operative presentation in clinic, defined as baseline. Age at presentation, sex, race, and ethnicity were demographic variables collected. Sport- and injury-related variables collected included competition level (school, club/select, college, or other), sport participation (single- or multi-sport athlete), sport participation volume (years in sport, days per week, weeks per year, weeks taken off per year), and recovery duration (days from surgery to return-to-sport clearance). PRO measures were also collected from the participant’s intake form or surveys administered through the Outcome Based Electronic Research Database (OBERD; Universal Research Solution, LLC; Columbia, Missouri), an electronic PRO system validated in youth (Sabatino et al., 2019). PROs included the AIMS, the Hospital for Special Surgery Pediatric Functional Activity Brief Scale (HSS Pedi-FABS), the Pediatric International Knee Documentation Committee Subjective Form (Pedi-IKDC), the Anterior Cruciate Ligament-Return to Sport after Injury scale (ACL-RSI), and the Athletic Coping Skills Inventory-28 (ACSI-28).

### Patient-Reported Outcome Measures

The AIMS is a validated measure for the young adult population that assesses athletic identity (Brewer et al., 1993), though it has not yet been validated for the youth population. The AIMS consists of 10 items rated on a Likert-type scale (1 = Strongly disagree, 7 = Strongly agree), and consists of three subscales including Social Identity (questions 1, 2, 3, and 7), Exclusivity (questions 4, 5, 6, and 9), and Negative Affectivity (questions 8 and 10). Social Identity is a measure of the extent of the athlete role dominating the social sphere; Exclusivity is a measure of the extent to which individuals both identify as athletes and do not identify with other roles; and Negative Affectivity is a measure of negative response to time away from sport. A higher score on the AIMS corresponds with a stronger identity as an athlete (Martin et al., 1997; Mitchell et al., 2021). Additionally, the AIMS may correlate with rehabilitation outcomes as described in Brewer et al. (2003) for individuals recovering from anterior cruciate ligament reconstruction (ACL-R).

The HSS Pedi-FABS is a functional activity scale developed to assess the participant’s activity level in the month prior to completing it and is a validated measure for the pediatric population (age 10-18; Fabricant et al., 2013a,b). The scale consists of eight items assessing how often the respondent runs, cuts, decelerates, or pivots along with assessing duration, endurance, competition level, and supervision. The eight items are scored on a scale of 0-30, with a higher score indicating the respondent being more active.

An additional PRO validated in the pediatric population (age 10-18) with knee disorders is the Pedi-IKDC (Kocher et al., 2011). The Pedi-IKDC assesses symptoms, function, and sport activity through 15 items on a scale of 0-100, where a higher score represents a higher level of function and lower level of symptoms (Nasreddine et al., 2017).

The ACL-RSI is a 12-item PRO created to assess the psychological readiness to return to play in athletes following an ACL-R and has been found to be a good predictor of pre-injury level of participation (Langford et al., 2009; Ardern et al., 2013). A higher score on the ACL-RSI indicates a higher confidence in returning to sport on a scale of 0-100.

The ACSI-28 is a validated measure to assess an athlete’s coping skills in seven domains: coachability, concentration, confidence and achievement motivation, coping, freedom from worrying, goal setting and mental preparation, and peaking under pressure (Webster et al., 2008). Each domain is evaluated on a scale of 0-12 and can be assessed independently; a total score can also be calculated ranging from 0-84. A higher score indicates greater strength in that domain and overall.

### Statistical Analysis

Two groups were created to determine differences in demographic variables, sport characteristics, and psychosocial measures by total AIMS score. Specifically, participants with high AIMS scores (AIMS_H_) were identified by a total score greater than 50, and low AIMS scores (AIMS_L_) were identified by a total score less than 50. Given the limited literature on the AIMS in a youth population, a threshold value of 50 was selected based on the average total score computed from this cohort. This value also corresponds to that chosen in Padaki et al., which investigates a comparable mean age (2018). Descriptive statistics (means and standard deviations) were calculated for each AIMS question and total score across all participants, as well as between the two AIMS groups. Independent samples t-tests and ANOVAs were performed to identify differences between equally distributed groups (e.g., male vs. female; AIMS_H_ vs AIMS_L_). A conventional 0.05 level of significance was set for all statistical tests.

## Results

Across all participants, the average total AIMS score was 49.4 ± 11.9 out of 70 total points (seven points per question). The total cohort identified the least with the athlete role on questions 6 (‘I need to participate in sport to feel good about myself’) and 9 (‘Sport is the only important thing in my life’). Alternatively, the cohort identified the most with the athlete role on questions 1 (‘I consider myself an athlete’), 2 (‘I have many goals related to sport’), and 3 (‘Most of my friends are athletes’). Between AIMS_H_ and AIMS_L_ groups, question 6 exhibited the greatest deviation (∼2.8 points; Table 2).

**TABLE 2:** Baseline AIMS total score and individual question ratings (mean ± SD) for all participants and both AIMS groups.

AIMS Question	AIMS Score		
	*AIMS*_*ALL*_	*AIMS*_*L*_	*AIMS*_*H*_
(1). I consider myself an athlete	6.5 ± 1.0	6.3 ± 1.3	6.8 ± 0.5
(2). I have many goals related to sport	5.9 ± 1.5	5.3 ± 1.8	6.5 ± 0.9
(3). Most of my friends are athletes	6.1 ± 1.4	5.6 ± 1.7	6.6 ± 0.8
(4). Sport is the most important part of my life	5.2 ± 1.8	4.1 ± 1.8	6.1 ± 1.2
(5). I spend more time thinking about sport than anything else	4.5 ± 1.9	3.3 ± 1.6	5.7 ± 1.3
(6). I need to participate in sport to feel good about myself	3.8 ± 2.3	2.3 ± 1.6	5.1 ± 1.9
(7). Other people see me mainly as an athlete	5.4 ± 1.8	4.4 ± 1.9	6.4 ± 1.0
(8). I feel bad about myself when I do poorly in sport	4.2 ± 2.2	3.1 ± 1.9	5.3 ± 1.8
(9). Sport is the only important thing in my life	2.8 ± 1.9	1.5 ± 0.9	3.9 ± 1.7
(10). I would be very depressed if I were injured and could not compete in sport	5.0 ± 2.2	3.7 ± 2.2	6.2 ± 1.3
**Total Score**	49.4 ± 11.9	39.4 ± 8.3	58.5 ± 5.7

AIMS total score at baseline significantly differed by sex (*p* = 0.002). Specifically, males exhibited higher AIMS scores by 4.8 points, and the AIMS_H_ group was found to be 58.5% male while the AIMS_L_ group was 61.1% female. Additionally, no differences in AIMS total score were found between any of the remaining demographic or sport-specific groupings. Alternatively, participants in the AIMS_H_ group reported ∼1.2 more ‘years active in sport’ (*p* = 0.049), a higher Pedi-FABS score (*p* = 0.038), and a lower ASCI-Coachability subscore (*p* = 0.027; Table 3). Notably, none of the three measures that exhibited AIMS group differences were found to significantly differ based on sex (*p* > 0.05).

**TABLE 3:** Differences in continuous measures between AIMS_H_ and AIMS_L_ groups.

Variable	AIMS_*L*_	AIMS_*H*_	
*Demographics/Participation Volume*	*Mean* ± *SD*	*Mean* ± *SD*	*p-value*
Age	15.9 ± 2.1	15.9 ± 2.0	0.974
Recovery Duration	266.8 ± 99.8	273.7 ± 101.9	0.706
Years Active in Primary Sport	6.3 ± 3.8	7.5 ± 3.5	**0.049**
Hours per Week	9.7 ± 5.7	11.7 ± 11.9	0.213
Days per Week	4.5 ± 1.6	4.8 ± 2.0	0.243
Weeks per Year	30.3 ± 16.3	30.9 ± 15.8	0.834
Weeks Off per Year	10.1 ± 11.4	10.4 ± 11.5	0.919
*Patient-Reported Outcome Measure*			
Pedi-FABS	18.9 ± 9.8	22.2 ± 8.8	**0.038**
Pedi-IKDC	48.5 ± 21.1	48.5 ± 23.3	0.985
ACL-RSI	54.7 ± 26.8	53.6 ± 24.2	0.815
ACSI-Coachability	10.5 ± 1.7	9.7 ± 2.3	**0.027**
ACSI-Concentration	8.9 ± 2.1	8.5 ± 2.1	0.344
ACSI-Confidence and Achievement Motivation	9.6 ± 1.9	9.7 ± 1.9	0.913
ACSI-Coping	8.0 ± 2.4	7.3 ± 2.5	0.093
ACSI-Freedom from Worry	7.5 ± 2.8	7.1 ± 3.0	0.420
ACSI-Goal Setting and Mental Preparation	6.5 ± 2.8	7.2 ± 2.9	0.203
ACSI-Peaking under Pressure	7.6 ± 2.9	8.1 ± 2.7	0.283
ACSI-Total	56.2 ± 16.3	57.6 ± 10.3	0.561

*Significance notated in bold.*

## Discussion

The current study is the first of our knowledge examining the relationship between demographics, sport participation, psychosocial self-reported measures, and athletic identity in a pediatric sports medicine practice. While the primary outcome measure, the AIMS, has not been validated in children, studies of similar populations in adolescents or young adults have produced comparable AIMS values among participants. Padaki et al. (2018) found average AIMS scores of 52.8 – 57.5 (depending on the study subgroup) in their analysis of both youth and young adult athletes. McKay et al. (2013) found average AIMS scores of 55.72 ± 7.54 in their population of youth hockey players while studying athletic identity and fear avoidance. Similarly, Brewer et al. (2003) found average AIMS scores of 44.16 ± 9.98 in their study of competitive and recreational athletes of ages 14-47 years old, and Manuel et al. (2002) found average AIMS scores of 47.20 ± 9.78 in their study of athletes with ages 15-18 years and injuries of varying severity. With an average AIMS score of 49.4 ± 11.9 in the current study, the AIMS appears to be an effective indicator of athletic identity in children.

This study found that athletes in this population rated themselves the highest on questions assessing their identification with the athlete role as their social identity (questions 1, 2, and 3), and lowest on questions assessing the degree to which their self-worth is exclusively derived from their athletic role (questions 6 and 9). One interpretation of this result is that while participants strongly identified with their role as an athlete, it was not their sole source of self-esteem, indicating that they have other facets of their identity outside of the athlete role. This finding is consistent with the lack of differences in training volume and specialization by AIMS score. Moreover, it is encouraging since recent literature has shown that well-rounded youth are the most resilient and cope more effectively with injury and transitions from sport because being an athlete is not their only source of confidence (Christino et al., 2015).

The current study identified differences in AIMS scores based on sex which may be accounted for by the sporting environment they live in. Many sports are dominated by male or female participation, and gender continues to be an underexplored factor in ACL research despite consistently higher ACL injury rates in females (Parsons et al., 2021). Differences based on race or ethnicity, age, sport type, or sport competition level were not observed. The literature lacks consensus on how these measures relate to athletic identity, though age and athletic identity may positively correlate (Lamont-Mills and Christensen, 2006; Anderson et al., 2009, 2011; Proios et al., 2012; Babić et al., 2015; Şekeroğlu, 2017; Piatt et al., 2018; Edison et al., 2021; Rae and Jenkins, 2021). Furthermore, the population examined was treated at a specialty sports medicine treatment center, possibly explaining the unequal distribution of competition levels. Future work on athletic identity should be conducted in larger, more diverse samples.

While various studies have suggested that training hours correlate to athletic identity, data from this cohort did not reflect differences in AIMS scores based on hours per week, days per week, or weeks per year (Piatt et al., 2018; Quinaud et al., 2020). Traditionally, those who participate in more elite levels of sport are thought to have higher athletic identity (Ahmadabadi et al., 2014). However, as we observed no differences in competition level, it appears that participating at elite levels of sport alone does not correlate with high athletic identity, in this population. Notably, years active in their primary sport was significantly different between AIMS_H_ and AIMS_L_. It seems that athletic identity is less determined by current sport competition level and time commitment, and more determined by the amount of time sport has been present in the formative years of life. For those who have played their sport since early childhood, it may be more likely that their closest friendships and personal connections are related to the sport, regardless of their participation level. While sport specialization has generally been associated with athletic identity, we observed no difference between single-sport and multi-sport athletes (Renton et al., 2021).

Furthermore, we found that those in the higher AIMS group scored lower in ACSI-Coachability, which is one of many important characteristics for successful recovery. Despite an incentive to return to sport, injury may be more damaging to the psyche of individuals with greater athletic identity, resulting in their lack of openness to suggestions in recovery, i.e., coachability. This finding may also be derived from the competitive, and sometimes individualistic, nature of many athletes. Some athletes may suffer greater psychological impact after injury by disregarding outside intervention and/or keeping to themselves as a coping mechanism (Christino et al., 2015). Still, no other ACSI measures were different by AIMS groups. Given the lack of differences identified in the remaining ACSI measures, additional work is needed to further investigate the relationship between athletic identity, coping mechanisms, and fear avoidance as it may be significant to injury outcomes (Fischerauer et al., 2018).

No differences in readiness as measured by ACL-RSI were observed between AIMS_H_ and AIMS_L_ groups despite previous studies suggesting differences may exist (Ardern et al., 2014, 2016; Fischerauer et al., 2018). Similarly, subjective knee function as measured by Pedi-IKDC was not different between groups. Both measures’ lack of differences is expected at recovery baseline as participants have not yet begun rehabilitation. However, activity as measured by Pedi-FABS scores did differ by AIMS group. Therefore, athletic identity and activity level may be correlated. While competition level did not see any differences in AIMS scores, activity level as measured by the Pedi-FABS could be a better representation of sport participation than competition level. Competition level may vary greatly between certain select/club level individuals – some may participate nationally at much higher intensity; others may participate in a local competitive league. Therefore, activity level should continue to be a focus of study with respect to athletic identity.

Limitations of the current study include a low sample size of certain demographic groups, specifically in race and ethnicity, and inconsistent sample sizes across variables. While some studies in young adults are available, this study was able to describe a population which had previously not been represented in the literature on athletic identity, and more specifically the AIMS. For this reason, a threshold of high and low AIMS scores has not been agreed upon in the literature. Thus, a threshold was chosen based on our cohort, which could be skewed given the region, type of treatment center, low sample size where testing was completed, and lack of healthy controls, though it appears consistent with other studies of similar populations. Additionally, by analyzing AIMS scores at baseline, only an initial assessment of the data was made in this study. Baseline in the current study is considered pre-rehabilitation; measurements at true baseline, or pre-injury, may yield different results. However, physicians and rehabilitation experts rarely see patients pre-injury, which suggests that the pre-rehabilitation baseline may be more clinically relevant.

The current study explores a population not previously studied with regards to athletic identity, sport participation, and psychosocial measures at a pediatric sports medicine practice. While youth athletes appear to be resilient, these results emphasize that they identify strongly with their role as an athlete and may suffer psychosocial consequences due to injury. Specifically, it was observed that sex, years active in sport, activity level, and coachability are important to consider at baseline before rehabilitation. Future work should validate the results in this study among a larger, more diverse population. Moreover, future studies should look to include assessments of the relationship between athletic identity, psychosocial factors, severity of diagnosis, and knee function over the course of recovery. Continuing research on such measures in athletic youths, thereby filling the current research gap, is essential to treat the debilitating effects of injury most effectively – both mentally and physically.

## Data Availability Statement

The raw data supporting the conclusions of this article will be made available by the authors, without undue reservation.

## Ethics Statement

The studies involving human participants were reviewed and approved by University of Texas Southwestern Institutional Review Board. Written informed consent from the participants’ legal guardian/next of kin was not required to participate in this study in accordance with the national legislation and the institutional requirements.

## Author Contributions

JM, SU, and ES contributed to conception and design of the study. JM and SU organized the database. SU performed the statistical analysis. JM wrote the first draft of the manuscript. HW and SU wrote sections of the manuscript. All authors contributed to manuscript revision, read, and approved the submitted version.

## Conflict of Interest

The authors declare that the research was conducted in the absence of any commercial or financial relationships that could be construed as a potential conflict of interest.

## Publisher’s Note

All claims expressed in this article are solely those of the authors and do not necessarily represent those of their affiliated organizations, or those of the publisher, the editors and the reviewers. Any product that may be evaluated in this article, or claim that may be made by its manufacturer, is not guaranteed or endorsed by the publisher.
